# Supplementary material for: Immune suppressive landscape in the human esophageal squamous cell carcinoma microenvironment
Source: Nat Commun. 2020 Dec 8;11:6268. doi: 10.1038/s41467-020-20019-0 (PMC7722722; doi:10.1038/s41467-020-20019-0)
Supplement: Supplementary file 1 — Supplementary Information [file 41467_2020_20019_MOESM1_ESM.pdf]

Immune suppressive landscape in the human esophageal squamous cell carcinoma microenvironment

Yingxia Zheng[1, 2, *, #], Zheyi Chen[1, *], Yichao Han [3, *], Li Han[1, *], Xin Zou[4], Bingqian Zhou[1], Rui Hu[5], Jie Hao[4], Shihao Bai[4], Haibo Xiao[5], Wei Vivian Li[6], Alex Bueker[7], Yanhui Ma[1], GuohuaXie[1], Junyao Yang[1], Shiyu Chen[1], Hecheng Li[3, #], Jian Cao[7, 8, #], Lisong Shen[1, 9, #]

[1]Department of Laboratory Medicine, Xin Hua Hospital, Shanghai Jiao Tong University School of Medicine, Shanghai, China; [2]Institute of Biliary Tract Diseases Research, Shanghai Jiao Tong University School of Medicine, Shanghai, China; [3]Department of Thoracic Surgery, Ruijin Hospital, Shanghai Jiao Tong University School of Medicine, Shanghai, China; [4]Key Laboratory of Systems Biomedicine (Ministry of Education), Shanghai Centre for Systems Biomedicine, Shanghai Jiao Tong University, Shanghai, China. [5]Department of Thoracic Surgery, Xin Hua Hospital, Shanghai Jiao Tong University School of Medicine, Shanghai, China. [6]Department of Biostatistics and Epidemiology, Rutgers School of Public Health, New Brunswick, NJ, USA. [7]Rutgers Cancer Institute of New Jersey, New Brunswick, NJ, USA. [8]Department of Medicine, Robert Wood Johnson Medical School, Rutgers University, New Brunswick, NJ, USA. [9]Faculty of Medical Laboratory Science,Shanghai Jiao Tong University School of Medicine, Shanghai, China.

[*]These authors contribute equally: Yingxia Zheng, Zheyi Chen, Yichao Han, Li Han.

[#]Correspondence e-mail: zhengyingxia@xinhuamed.com.cn; lihecheng2000@hotmail.com; jian.cao@cinj.rutgers.edu; lisongshen@hotmail.com

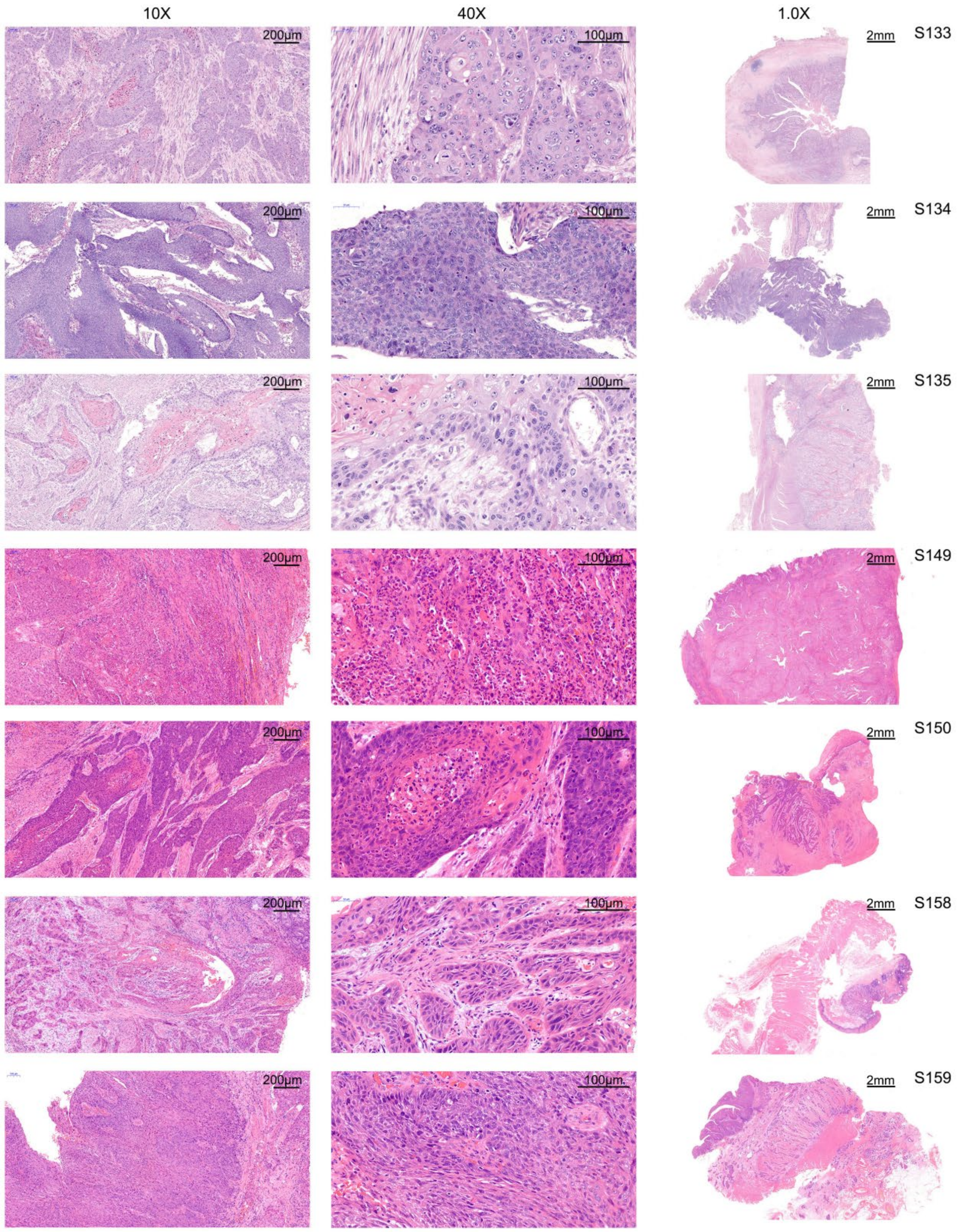

Supplementary Fig.1 IHC staining of seven ESCC tumor tissues.

HE staining showed tumor cells of seven patients with ESCC, showed the 1.0x, 10x and 40X, respectively. Representative field from each patient was shown.

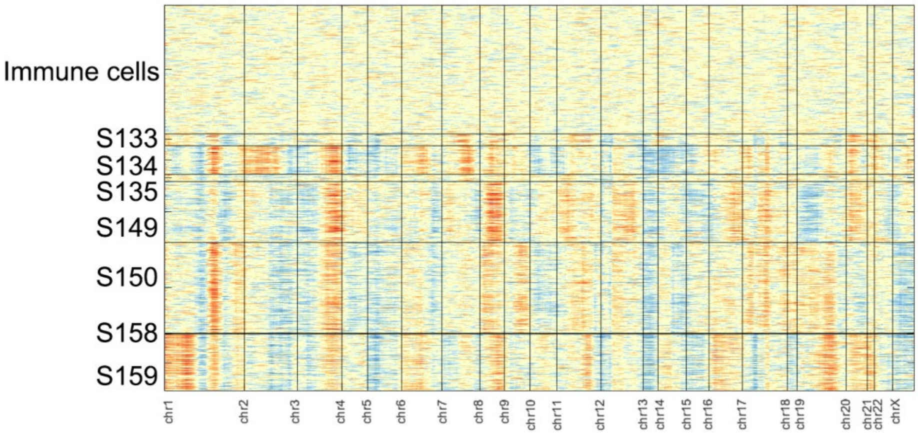

Supplementary Fig.2 Chromosomal landscape of inferred large-scale copy number variations (CNVs) distinguishes malignant (lower panel) from immune cells (up panel). The “other” cell cluster from tumors was shown with individual cells (y-axis) and chromosomal regions (x-axis). Amplifications (red) or deletions (blue) were inferred by averaging expression over 100-gene stretches on the respective chromosomes.

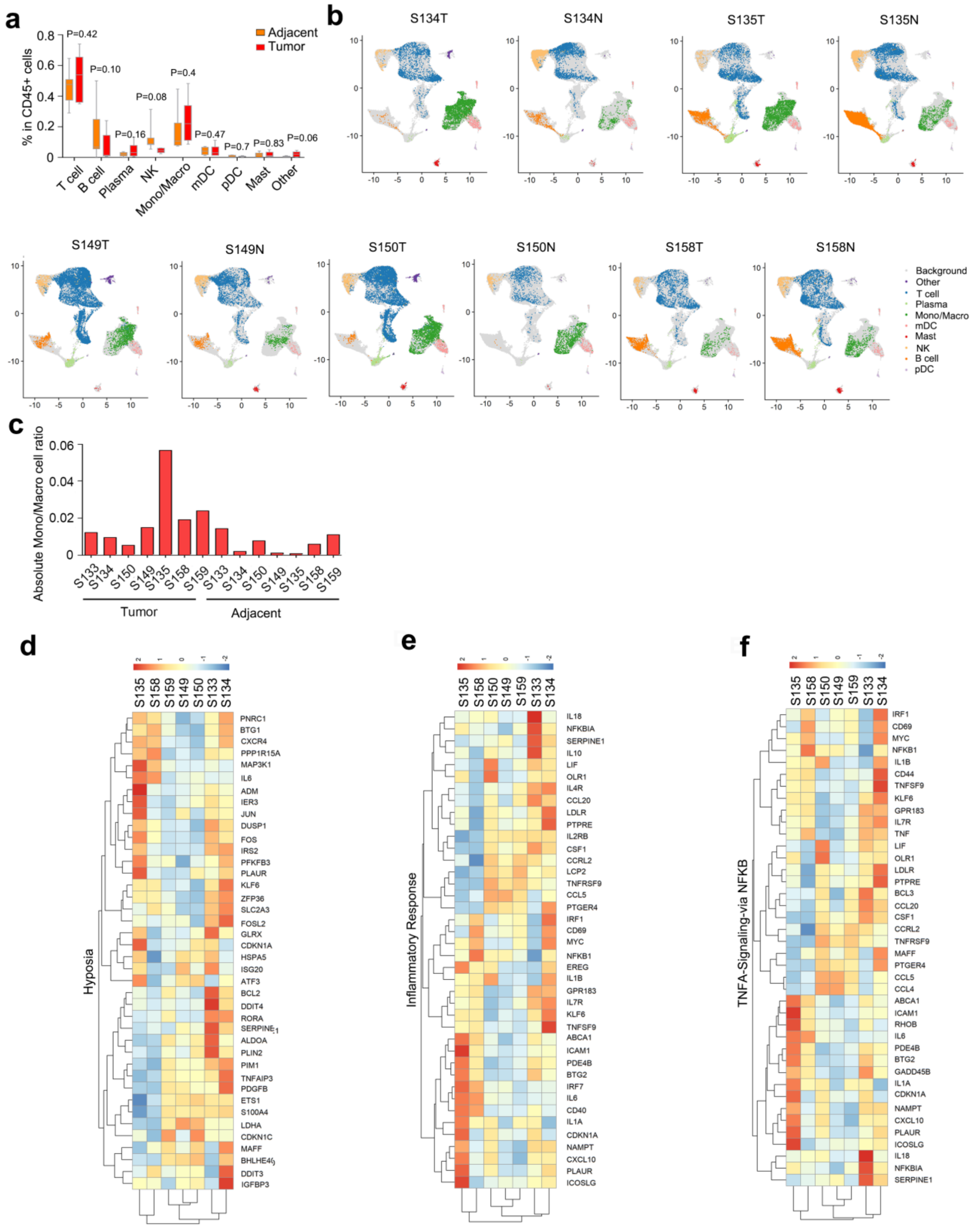

Supplementary Fig. 3 Additional details on heterogeneity of immune systems of ESCC, related to Fig. 1

(a) Bar plot showed each cell type between tumor and adjacent tissue (n=7). Each box represented the interquartile range (IQR, the range between the 25th and 75th

percentile) with the mid-point of the data, whiskers indicated the upper and lower value. *P* value was calculated by two-tailed paired Student's t-test. (**b**) UMAP plot of complete immune systems from 7 ESCC tumor and adjacent tissues, representative of S134, S135, S149, S150 and S158. Cell colored by clusters and labeled with inferred cell types. (**c**) The absolute cell ratio of monocytes/macrophage in total cells of the 14 samples. (**d-f**) Expression of Hallmark signatures: hypoxia (**d**), inflammatory response (**e**), TNFA-signaling via NFKB (**f**), across immune cells from each patient, displaying Z-scored mean expression of genes in each signature. *P* value of signatures about hypoxia, inflammation response and TNFA-via NFKB pathways between S135&S158 vs S133&S134 was $9.9e^{-76}$, $4.3e^{-37}$, and $4.0e^{-18}$, respectively. Wilcoxon rank sum test.

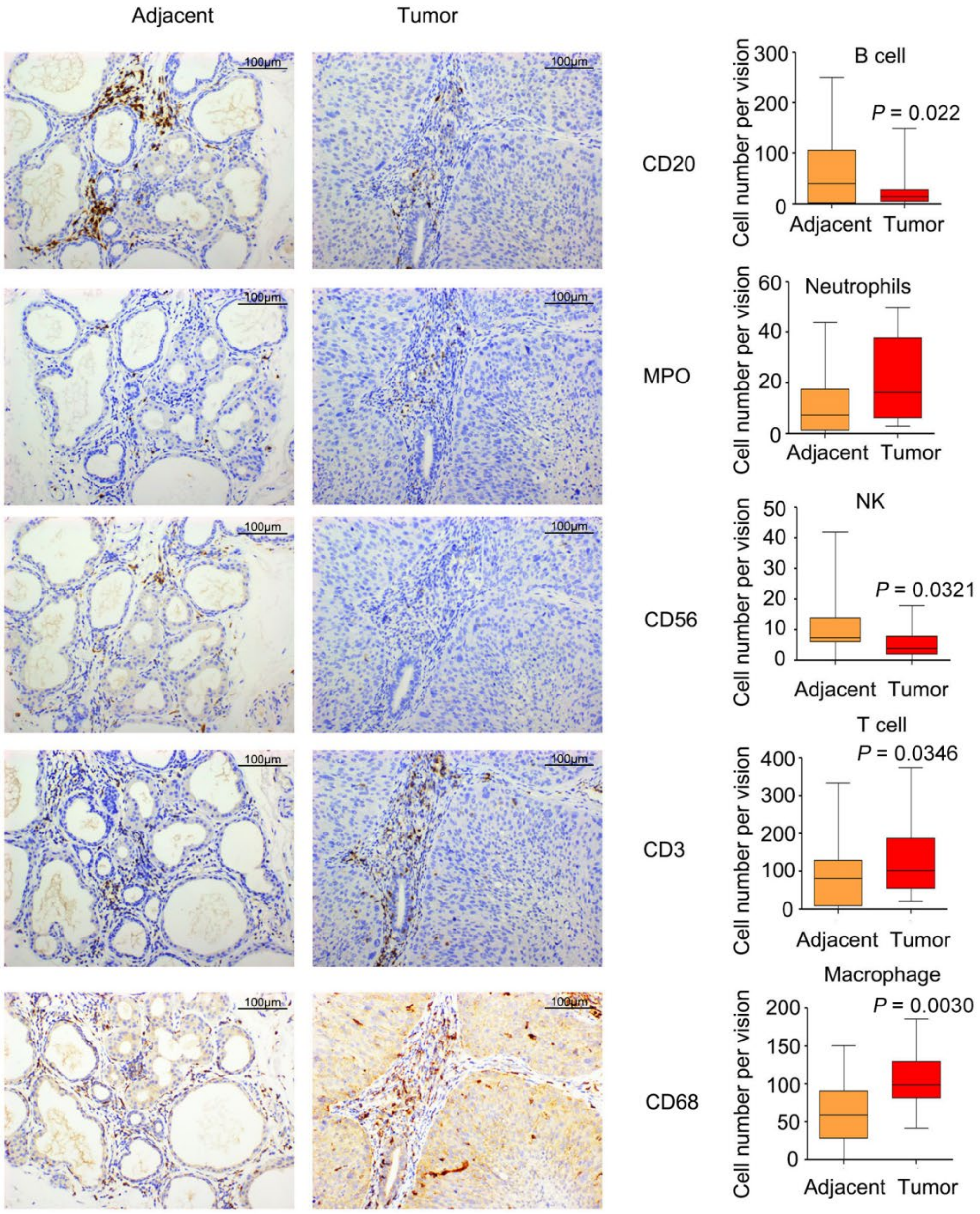

Supplementary Fig. 4 Additional details on heterogeneity of immune systems of ESCC, related to Fig. 1

IHC stained of CD3 (T cell), CD56 (NK), CD20 (B cell), CD68 (macrophage), MPO (neutrophil) in adjacent and tumor tissue of ESCC (n=11), and analysis of positive of cell number in each field. Representative field from each patient was shown. Each box represented the interquartile range (IQR, the range between the 25th and 75th

percentile) with the mid-point of the data, whiskers indicated the upper and lower value. *P* value was calculated by two-tailed Student's *t*-test.

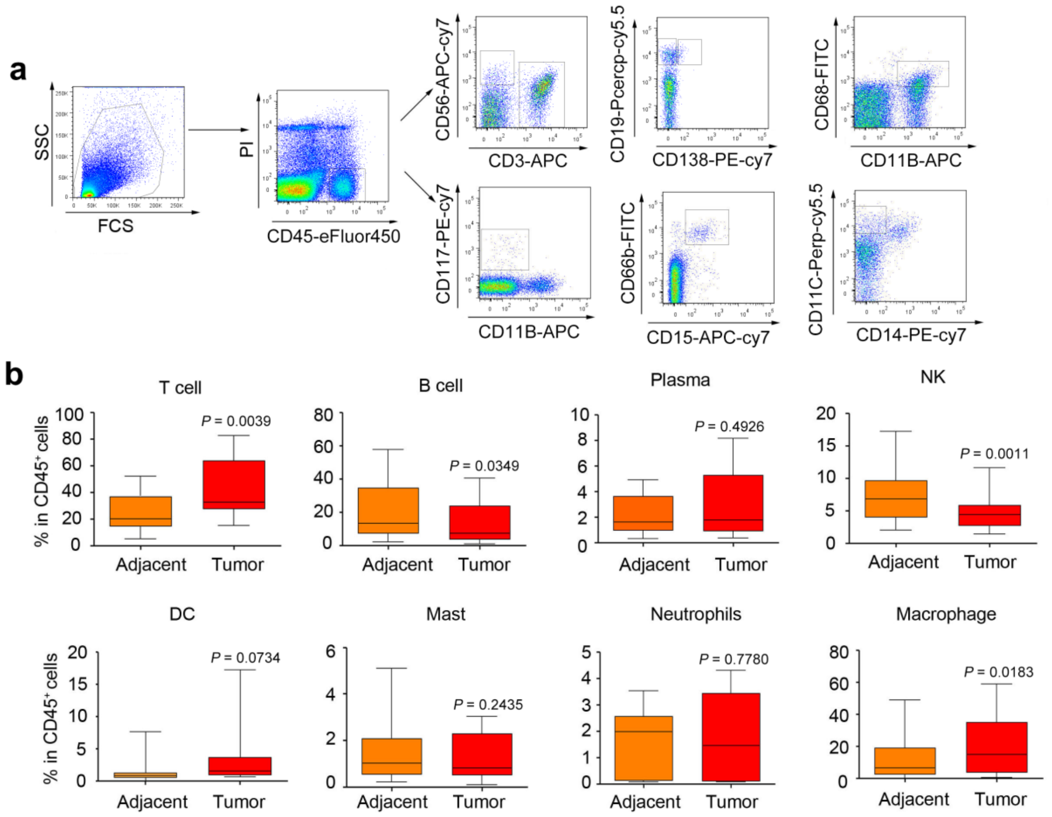

Supplementary Fig. 5 Additional details on heterogeneity of immune systems of ESCC, related to Fig. 1

FACS analysis of adjacent and ESCC tumor tissues (n=11) of immune subsets, including T cells, B cells, plasma, NK, DC, mast, neutrophils, and macrophages, the gating strategies (**a**) and percentages (**b**) were shown. Each box represented the interquartile range (IQR, the range between the 25th and 75th percentile) with the mid-point of the data, whiskers indicated the upper and lower value. *P* value was calculated by two-tailed Student's *t*-test.

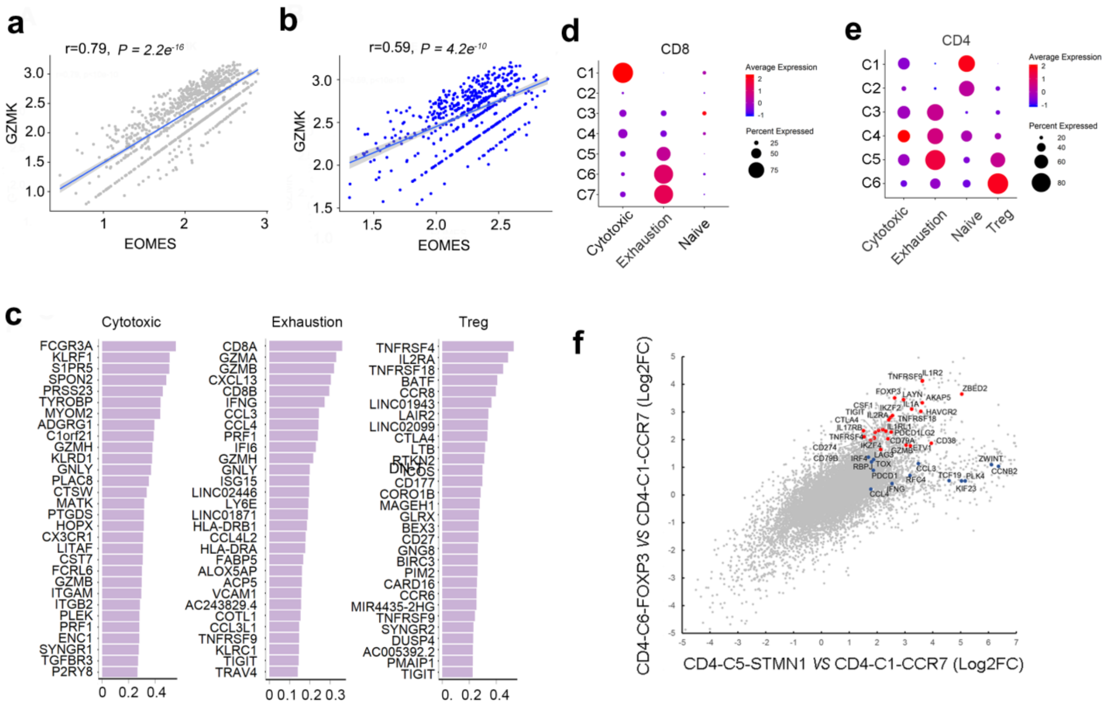

Supplementary Fig. 6 T cells characterization in ESCC, related to Fig. 2

(**a, b**) The correlation of EOMES and GZMK in CD8-C3-GZMK (**a**) and total CD8 T cells (**b**). The *P and r* value represented Pearson's correlation and its coefficient of determination. (**c**) Bar graph showing the top 30 genes that are most correlated with FGFBP2 (left panel) and LAG3 (middle panel) across CD8+ T cells. Bar graph showing the top 30 genes that are most correlated with FOXP3 (right panel) across CD4+ T cells. (**d**) Dot plot of representative cytotoxic, exhaustion, and naive signatures in CD8 T cell clusters, Z-score normalized log2 (count+1). (**e**) Dot plot of representative cytotoxic, exhaustion, naïve, and Treg signatures in CD4 T cell clusters, Z-score normalized log2 (count+1). (**f**) Gene enrichment of CD4-C5-STMN1 CD4 exhausted T cells and CD4-C6-FOXP3 Treg cells over CD4-C1-CCR7 naive cells. Showing data for strongly expressed genes (mean molecules per cell > = 0.05) and labeling genes of interest and strongly enriched genes.

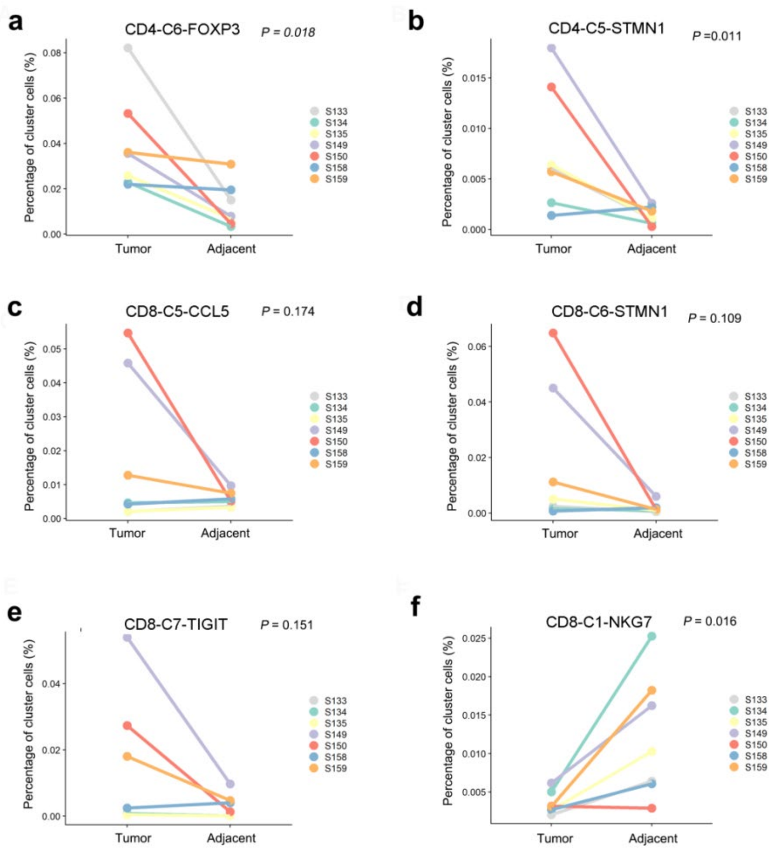

Supplementary Fig. 7 Altered status of T and NK cells in tumors, related to Fig. 3

Percentages of related clusters in tumor and adjacent tissues (n=7). Dot colors represent different samples. *P* value was calculated by two-tailed paired Student's *t*-test.

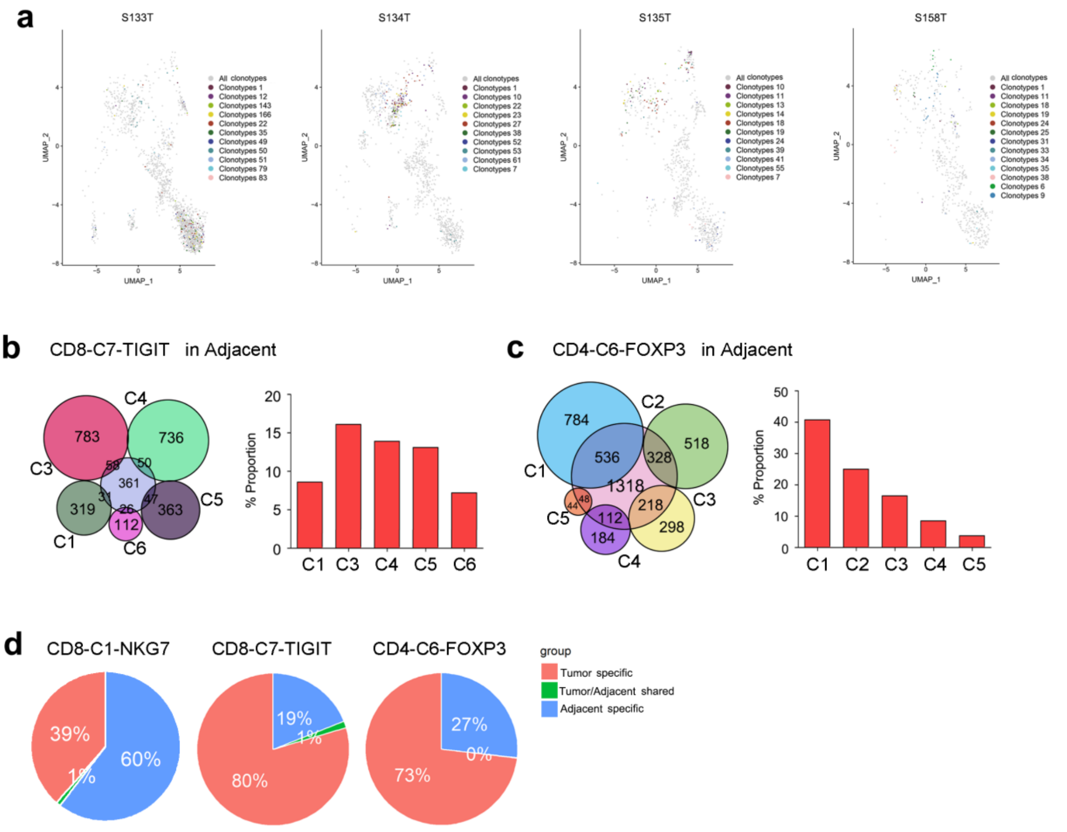

Supplementary Fig. 8 Single cell TCR sequencing, related to Fig. 4

(a) Representative examples of dominant clonotypes (top ten in color) from each tumor (gray) identified by TCR sequencing. (**b**)The number (left panel) and percentage (right panel) of shared TCRs between CD8-C7-TIGIT and other CD8 clusters in adjacent tissues. (**c**) The number (left panel) and percentage (right panel) of shared TCRs between CD4-C6-FOXP3 and other CD4 clusters in adjacent tissues. (**d**) The distribution of CD8-C1-NKG7, CD8-C7-TIGIT, CD4-C6-FOXP3 cell clonotypes between tumor and adjacent tissues.

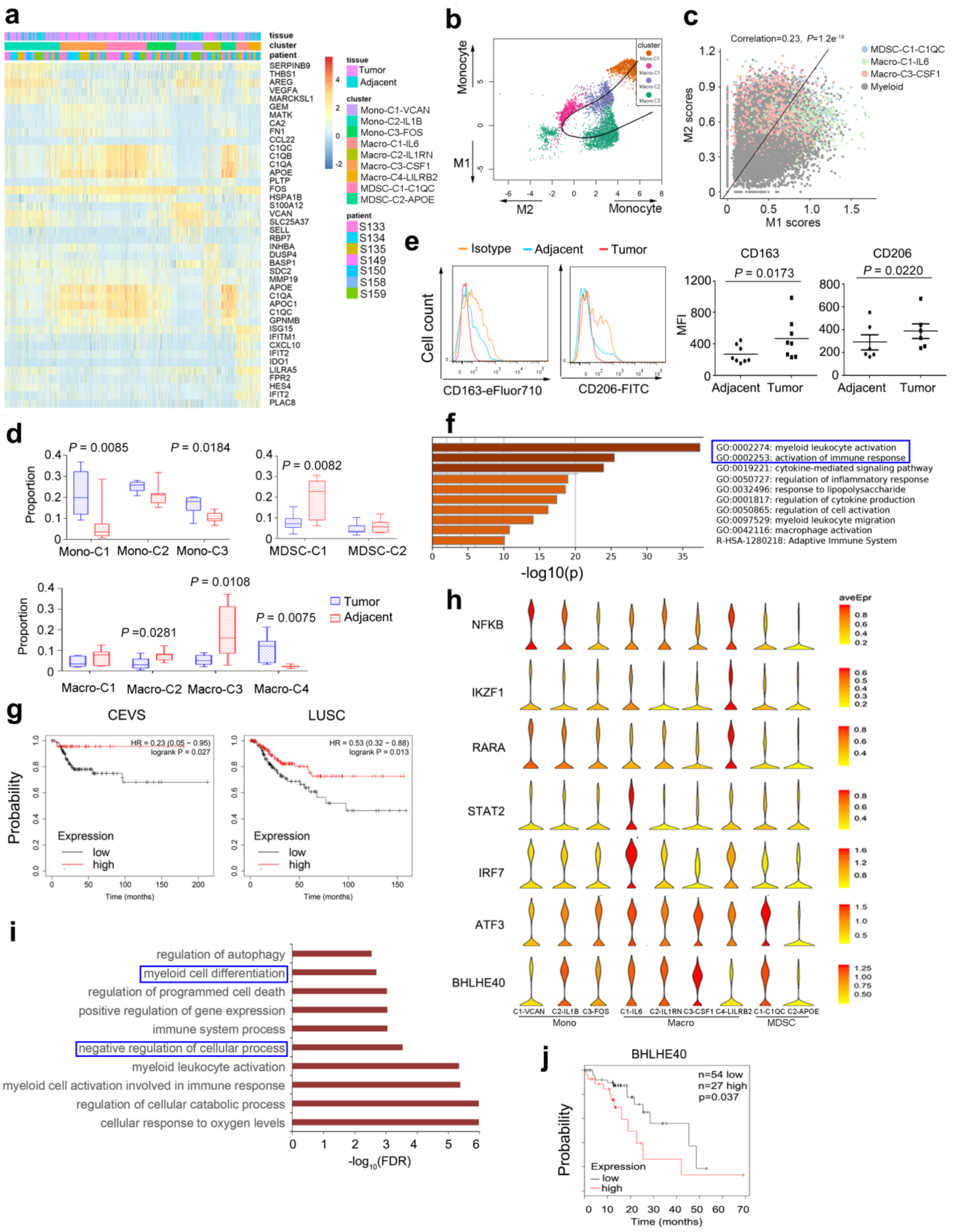

Supplementary Fig. 9 Detailed characterization of myeloid cells, related to Fig. 5.

(a) Heatmap of the cluster marker genes. The top bar indicates the origins, the middle bar indicates the clusters, and the lower bar indicates the different patients. The color density indicated expression level, Z-score normalized log2 (count+1). (**b**) The

trajectory of Macro-C3-CSF1, Mono-C1-VCAN, Macro-C2-IL1RN, Macro-C1-IL6 state transition in a two-dimensional state-space inferred by Slingshot. Each dot corresponds to one single-cell, colored according to its cluster label. Arrows show the increasing directions of certain cell properties. (**c**) Scatterplot of normalized mean expression of M1 and M2 signature per cell; cells assigned to myeloid clusters were colored by cluster type. Z-score normalized log2 (count+1). The *P* and r value represents Pearson's correlation and its coefficient of determination. (**d**) Box plot of each monocytes/macrophage cluster between the tumor and adjacent tissue (n=7). Each box represented the interquartile range (IQR, the range between the 25th and 75th percentile) with the mid-point of the data, whiskers indicated the upper and lower value. *P* value was calculated by two-tailed paired Student's *t*-test. (**e**) Flow cytometry measured CD68+ macrophage CD163 (n=8) and CD206 (n=6) expression in ESCC and adjacent tissues. Data were presented as mean ±SEM; *P* value was calculated by two-tailed Student's *t*-test. (**f**) Bar graph of enriched terms generated in the Metascape website across the input gene lists generated in **Fig. 5d** Turquoise module. The blue boxes indicated the pathways were highlight. (**g**) Kaplan-Meier overall survival curves of TCGA CEVS and LUSC patients with top 50 genes generated in **Fig.5f**. *P*-values were calculated using the two-tailed log-rank test. **(h)**Violin plots showing the average expression value of a given gene in each cluster. The expression is measured as the log2 (count+1). (**i**) Bar graph of enriched terms of BHLHE40 gargets genes generated by SCENIC of **Fig.5h**. The blue boxes indicated the pathways were highlight. (**j**) Kaplan-Meier overall survival curves of TCGA ESCC patients with high or low expression of BHLHE40, *P* value was calculated using the two-tailed log-rank test.

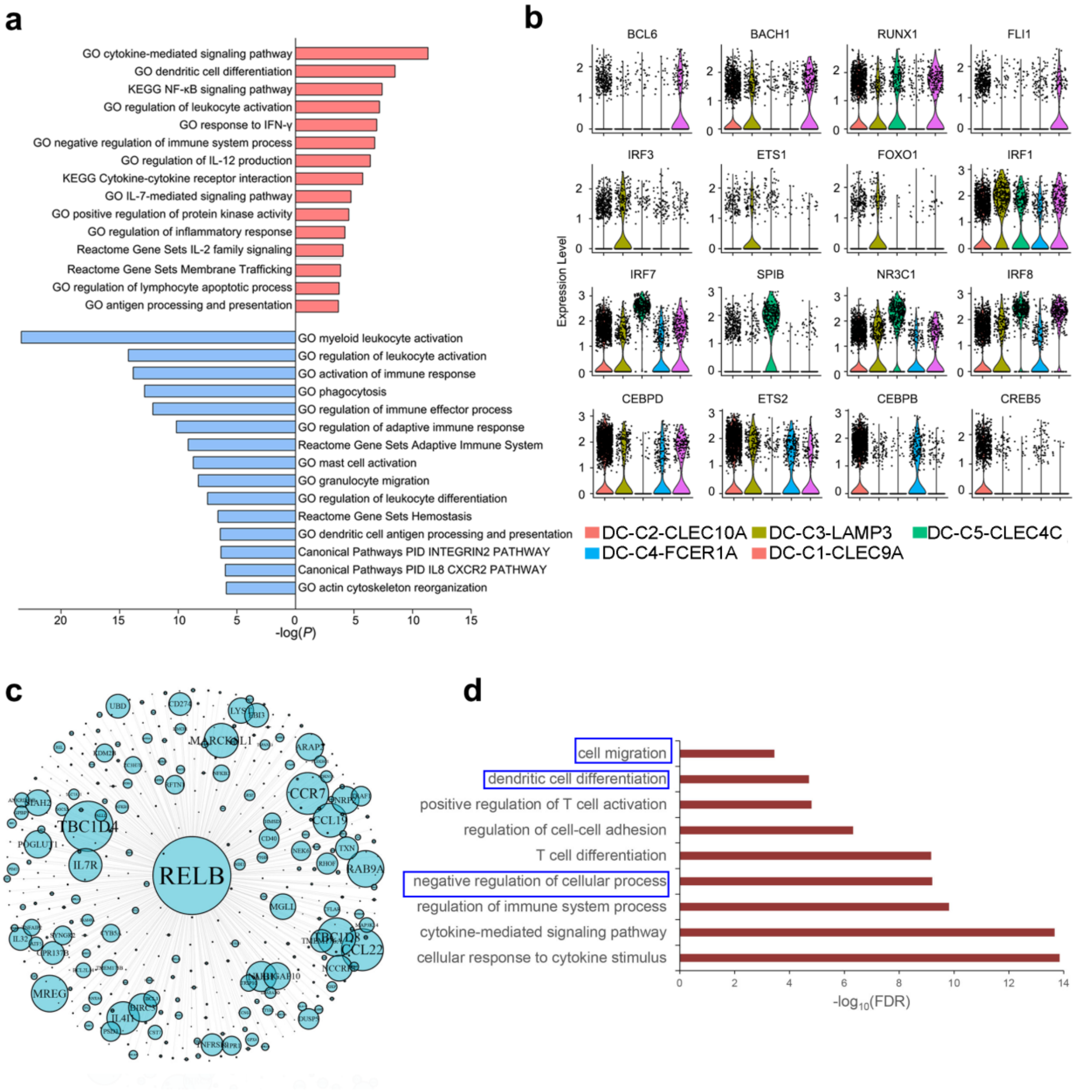

Supplementary Fig. 10 Detailed characterization of myeloid cells, related to Fig. 6

(a) Bar graph of enriched terms generated in the Metascape website across the input gene lists generated in **Fig. 6d**, red bar represented upregulation, and the blue bar represented downregulation. (**b**) Violin plots comparing the indicated gene expression in DC cell clusters. The expression was measured as the log2 (count+1). (**c**) Network analysis of transcription factor RELB and its targets through iGraph, the size of the

circle represents the co-expression relationship score. (**d**) Bar graph of enriched terms of RELB targets genes generated by SCENIC of supplementary **Fig.8c**. The blue boxes indicated the pathways were highlight.

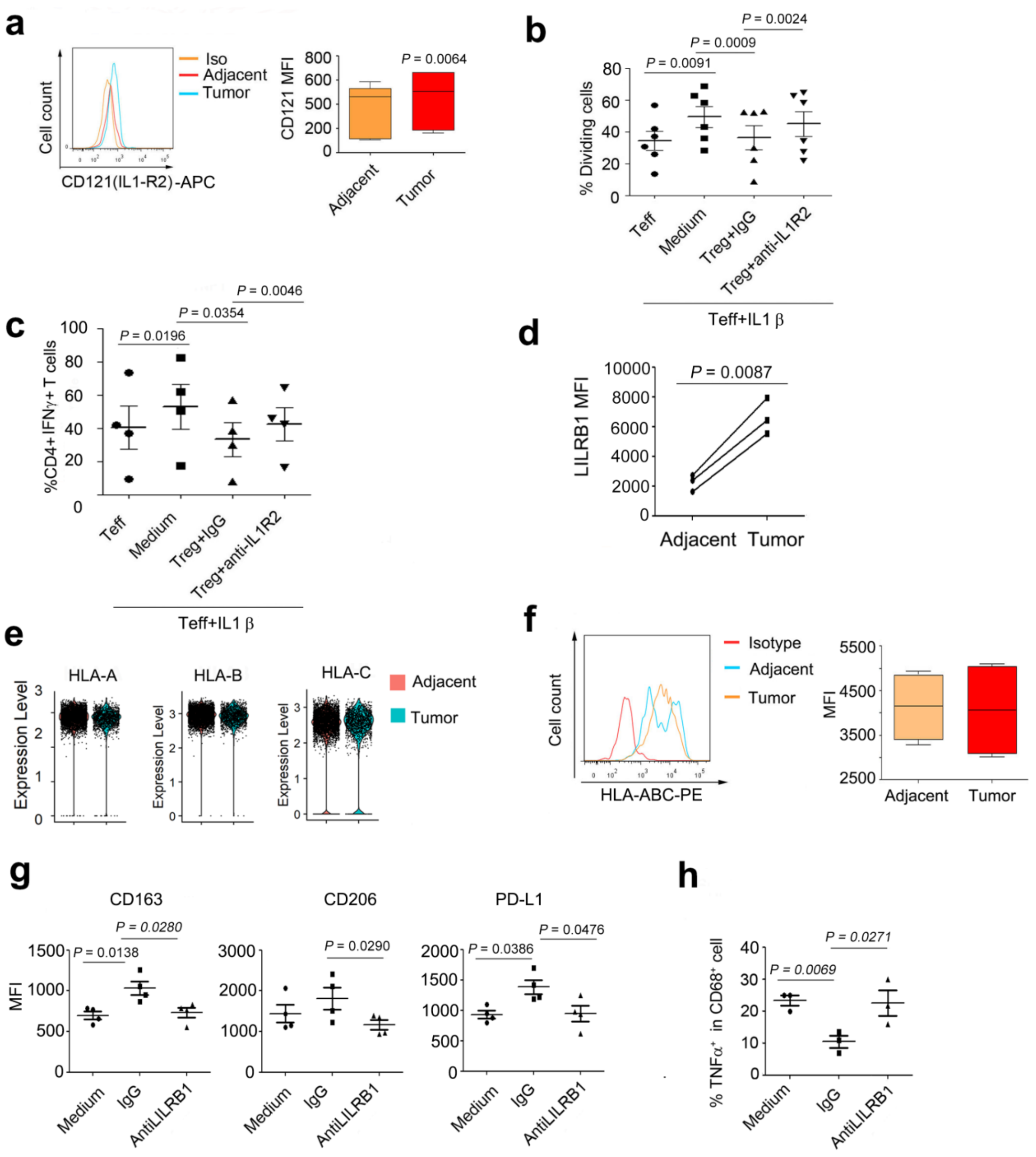

Supplementary Fig.11 Additional details on Ligand-Receptor-based interaction between immune cells in ESCC related to Fig.7

(**a**) FACS analysis of IL1R2 expression in Treg cells from adjacent and ESCC tumor tissues (n=5). Each box represented the interquartile range (IQR, the range between the 25th and 75th percentile) with the mid-point of the data, whiskers indicate the upper and lower value. *P* value was calculated by two-tailed Student's *t*-test. (**b**) Bar

plot showed Teff dividing cells in Fig.7e (n=6), data were presented as the mean ± SEM, *P* value was calculated by two-tailed Student's *t*-test. **(c)** Bar plot showed Teff expressing FNγ cell in Fig.7**f** (n=4), data were presented as the mean ±SEM; *P* value was calculated by two-tailed paired Student's *t*-test. (**d**) Dot plot showed macrophage cells LILRB1 expression mean fluorescence intensity (MFI) in tumor and adjacent tissue in Fig.7i, (n=3), data were presented as the mean ±SEM; *P* value was calculated by two-tailed Student's *t*-test. (**e**) Violin plot showed the HLA-A, HLA-B, HLA-C average expression in Treg cells from tumor and adjacent tissues. The expression was measured as the log2 (count+1). (**f**) FACS analysis of HLA-A, B, C expression in Treg cell from adjacent and ESCC tumor tissues (n=5). Each box represented the interquartile range (IQR, the range between the 25th and 75th percentile) with the mid-point of the data, whiskers indicated the upper and lower value. (**g**) Bar plot showed macrophage cells expressing CD163, CD206, PDL1 in Fig.7**k** (n=4), data were presented as the mean ±SEM; *P* value was calculated by two-tailed Student's *t*-test. (**h**) Bar plot showed macrophage cells expressing TNFα in Fig.7**l** (n=3), data were presented as the mean ±SEM; *P* value was calculated by two-tailed Student's *t*-test.

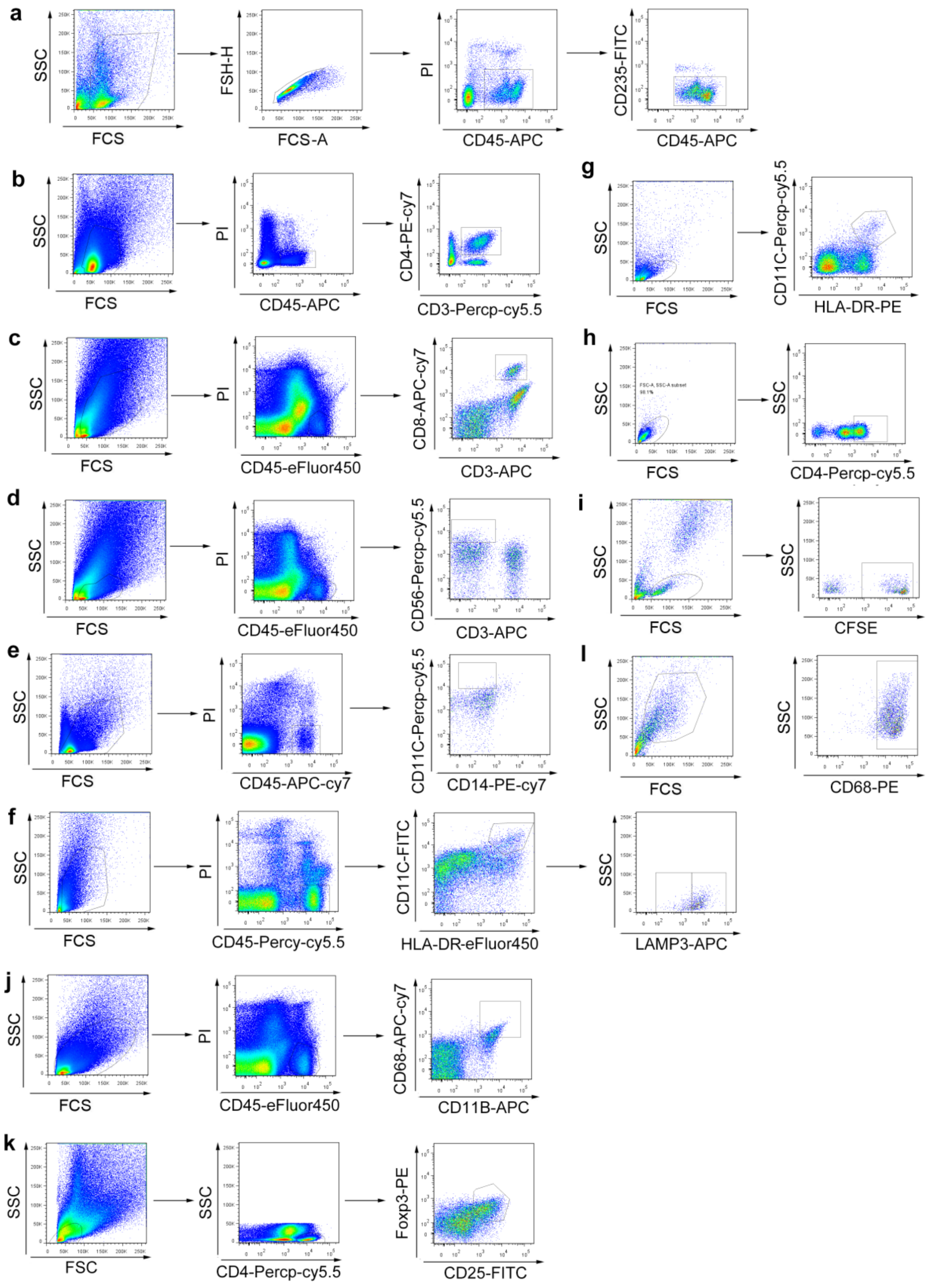

Supplementary Fig.12 FACS sequential gating/sorting strategies.

(**a**) The CD45+ CD235- PI- cells sorting strategy; (**b-d**)The Fig. **3c**, **f** and **i** gating strategies; (**e**) The Fig. **6e** gating strategy; (**f**) The Fig. **6f** gating strategy; (**g**)The Fig. **6h** gating strategies; (**h**)The Fig. **6i** gating strategy; (**i**)The Fig. **7e** and **f** gating

strategies; (**j**)The Fig. **7i**, Supplementary Fig.**9e** gating strategies; (**k**) The Supplementary Fig.**11a** and **f** gating strategies; (**l**) The Fig. **7k** and **l** gating strategies.

Supplementary Table 1: sample information

The number of patients are 28 (7 scRNA-seq, 21 Flow analysis); age ranged from 53 to 81 (median 67); only 2 females.

Patient ID	Clinical Information			Analysis	Sequencing Statistics				
	Pathology	Stage	Tissue		Estimated Number of Cells	Mean Reads per Cell	Median Genes per Cell	Total Genes Detected	Sequencing
S133	ESCC	IIIB	Primary Tumor	scRNA-seq	12342	28019	1082	23006	73.20%
			Solid Tissue Normal	scRNA-seq	6134	55113	816	20465	90.9
S134	ESCC	IIIA	Primary Tumor	scRNA-seq	8338	43536	935	21923	82.80%
			Solid Tissue Normal	scRNA-seq	5057	66636	1070	20000	91.60%
S135	ESCC	IIA	Primary Tumor	scRNA-seq	11018	43717	874	21576	86.70%
			Solid Tissue Normal	scRNA-seq	10680	36121	1084	21127	84.70%
S149	ESCC	IIA	Primary Tumor	scRNA-seq	10727	35086	1428	23574	77.60%
			Solid Tissue Normal	scRNA-seq	4375	82970	1272	21612	91.00%
S150	ESCC	IIIB	Primary Tumor	scRNA-seq	15820	24914	893	22003	83.70%
			Solid Tissue Normal	scRNA-seq	3502	126381	975	19463	95.90%
S158	ESCC	IIB	Primary Tumor	scRNA-seq	4986	70218	1026	19857	81.20%
			Solid Tissue Normal	scRNA-seq	6597	55469	1285	21268	86.20%
S159	ESCC	IIIB	Primary Tumor	scRNA-seq	7325	56195	1034	21379	86.30%
			Solid Tissue Normal	scRNA-seq	8256	44969	1051	21866	86.40%

Patient ID	Pathology	Stage	Analysis
E1	ESCC	IIA	Flow Cytometry
E2	ESCC	IIA	Flow Cytometry
E3	ESCC	IIIB	Flow Cytometry
E4	ESCC	IIA	Flow Cytometry
E5	ESCC	IIIB	Flow Cytometry
E6	ESCC	IIIA	Flow Cytometry
E7	ESCC	IIA	Flow Cytometry
E8	ESCC	IIA	Flow Cytometry
E9	ESCC	IIA	Flow Cytometry
E10	ESCC	IVA	Flow Cytometry
E11	ESCC	IIA	Flow Cytometry
E12	ESCC	IIIB	Flow Cytometry
E13	ESCC	IIA	Flow Cytometry
E14	ESCC	IIIB	Flow Cytometry
E15	ESCC	IIA	Flow Cytometry
E16	ESCC	IIIB	Flow Cytometry
E17	ESCC	IIIB	Flow Cytometry
E18	ESCC	IIIB	Flow Cytometry
E19	ESCC	IIIB	Flow Cytometry
E20	ESCC	IIA	Flow Cytometry
E21	ESCC	IIIB	Flow Cytometry

Supplementary Table 2: antibody information

Flow antibodies:

Specificity	Fluorophore	Clone	Manufacturer	Cat. No
CD117 (c-kit)	PE/Cyanine7	104D2	Biolegend	313212
CD11b	APC	CBRM1/5	eBioscience	17-0113-42
CD11c	PerCP/Cyanine5.5	3.9	Biolegend	301623
CD11c	FITC	3.9	eBioscience	11-0116-42
CD138 (Syndecan-1)	PE/Cyanine7	DL-101	eBioscience	25-1389-42
CD14	PE/Cyanine7	M5E2	BD Pharmingen	561385
CD15 (SSEA-1)	APC/Cyanine7	W6D3	Biolegend	323048
CD159a (NKG2A)	FITC	REA110	Miltenyi Biotec	130-113-565
CD163	PerCP/eFluor 710	eBioGHI/61 (GHI/61)	eBioscience	46-1639-42
CD19	PerCP/Cyanine5.5	HIB19	Biolegend	302230
CD197 (CCR7)	PE/Cyanine7	G043H7	Biolegend	353225
CD206 (MMR)	Alexa Fluor 488	19.2	eBioscience	53-2069-42
CD235ab	FITC	HIR2	Biolegend	306610
CD25	FITC	M-A251	Biolegend	356106
CD274 (PD-L1)	BV421	MIH1	BD Horizon	563738
CD279 (PD-1)	PerCP/Cyanine5.5	EH12.2H7	Biolegend	329913
CD3	APC	HIT3a	Biolegend	300312
CD3	PerCP/Cyanine5.5	HIT3a	Biolegend	300327
CD4	PerCP/Cyanine5.5	OKT4	Biolegend	317428
CD4	PerCP/Cyanine5.5	A161A1	Biolegend	357409
CD45	eFluor 450	2D1	eBioscience	48-9459-42
CD45	APC	HI30	BD Pharmingen	560973
CD45	APC/Cyanine7	HI30	Biolegend	304014
CD45	PerCP/Cyanine5.5	2D1	Biolegend	368503
CD56 (NCAM)	APC/Cyanine7	HCD56	Biolegend	318332
CD56 (NCAM)	PE/Cyanine5.5	CMSSB	eBioscience	35-0567-42
CD63 (LAMP-3)	Alexa Fluor 647	H5C6	BD Pharmingen	561983
CD66b	FITC	G10F5	Biolegend	305104
CD68	FITC	Y1/82A	Biolegend	333805
CD68	APC/Cyanine7	Y1/82A	Biolegend	333822
CD68	PE	Y1/82A	eBioscience	12-0689-42
CD8	APC/Cyanine7	SK1	Biolegend	344714
CD83	APC/Cyanine7	HB15e	Biolegend	305330
FOXP3	eFluor 450	236A/E7	eBioscience	48-4777-42
FOXP3	PE	259D/C7	BD Pharmingen	560046
HLA-ABC	PE	W6/32	eBioscience	12-9983-42
HLA-DR	eFluor 450	L243	eBioscience	48-9952-42
HLA-DR	PE	L243	Biolegend	307605
IDO	FITC	eyedio	eBioscience	11-9477-42
IFN-γ	BV421	B27	BD Horizon	562988
IL-1 RII	APC	34141	R&D Systems	FAB663A
TNF-a	APC	MAb11	eBioscience	17-7349-82

Specificity	Fluorophore	Clone	Manufacturer	Cat. No
LILRB1		Polyclonal	R&D Systems	AF2017
Donkey Anti-Goat IgG	NorthernLights 637		R&D Systems	NL002

Stimulation or blocking antibodies:

Specificity	Manufacturer	Cat. No	Clone
CD3	eBioscience	16-0037-85	OKT3
CD28	eBioscience	16-0289-85	CD28.2
IL-1 RII	R&D Systems	MAB663-100	34141
LILRB1	R&D Systems	AF2017	Polyclonal
Mouse IgG2a isotype	Invitroen	16-4724-82	Ebm2a

Multi-color immunohistochemistry antibodies:

Specificity	Manufacturer	Cat. No	Clone	Host
CD4	Abcam	ab181724	EPR6855	R
FOXP3	abcam	ab22510	Polyclonal	M
LILRB1	Abcam	ab249583	EPR11256	R
CD68	Abcam	ab955	KP1	M
CD11c	Abcam	ab216655	EP1347Y	R
IDO	Abcam	ab245737	SP260	R
PD-L1	CST	13684S	E1L3N	R
LAMP3	Abcam	ab111090	Polyclonal	R

Immunohistochemistry antibodies:

Specificity	Manufacturer	Cat. No	Clone	Host
CD20	LEICA	NCL-L-CD20-L26	L26	M
CD3	LEICA	NCL-L-CD3-565	LN10	M
Myeloperoxidase (MPO)	Long island	0405	EP151/SP72	R
CD56	Long island	0148	56C04	M
CD68	Long island	0160	KP1/PG-M1	M
