# Supplementary material for: Immune suppressive landscape in the human esophageal squamous cell carcinoma microenvironment
Source: Nat Commun. 2020 Dec 8;11:6268. doi: 10.1038/s41467-020-20019-0 (PMC7722722; doi:10.1038/s41467-020-20019-0)
Supplement: Supplementary file 3 — Description of Additional Supplementary Files [file 41467_2020_20019_MOESM3_ESM.pdf]

**Description of Additional Supplementary Files**

File Name: Supplementary Data 1
Description: The ratios of each immune cell type to all cells analyzed by flow cytometry.

File Name: Supplementary Data 2
Description: The top differentially expressed genes of each cluster for T and NK cells.

File Name: Supplementary Data 3
Description: The naïve, Treg, exhuastion, and cytotoxic genes signatures.

File Name: Supplementary Data 4
Description: The top differentially expressed genes of each cluster for myeloid cells.

File Name: Supplementary Data 5
Description: The gene signatures of monocytes, classically activated macrophages (M1), alternatively activated macrophages (M2) and myeloid-derived suppressor cells (MDSCs)

File Name: Supplementary Data 6
Description: Top 50 genes that most correlated between Turquoise module and Mono-C1-VCAN cluster.

File Name: Supplementary Data 7
Description: Macrophages provide ligand, the Macrophages and Tregs interactions score using scTHI.
