# Supplementary material for: Immune suppressive landscape in the human esophageal squamous cell carcinoma microenvironment
Source: Nat Commun. 2020 Dec 8;11:6268. doi: 10.1038/s41467-020-20019-0 (PMC7722722; doi:10.1038/s41467-020-20019-0)
Supplement: Supplementary file 11 — Reporting Summary [file 41467_2020_20019_MOESM11_ESM.pdf]

nature research

Corresponding author(s): Yingxia Zheng, Hecheng Li, Jian Cao, and Lisong Shen

Last updated by author(s): Oct 19, 2020

Reporting Summary

Nature Research wishes to improve the reproducibility of the work that we publish. This form provides structure for consistency and transparency in reporting. For further information on Nature Research policies, see Authors & Referees and the Editorial Policy Checklist .

Statistics

For all statistical analyses, confirm that the following items are present in the figure legend, table legend, main text, or Methods section.

n/a	Confirmed	
☐	☒	The exact sample size (*n*) for each experimental group/condition, given as a discrete number and unit of measurement
☐	☒	A statement on whether measurements were taken from distinct samples or whether the same sample was measured repeatedly
☐	☒	The statistical test(s) used AND whether they are one- or two-sided<br>*Only common tests should be described solely by name; describe more complex techniques in the Methods section.*
☒	☐	A description of all covariates tested
☐	☒	A description of any assumptions or corrections, such as tests of normality and adjustment for multiple comparisons
☐	☒	A full description of the statistical parameters including central tendency (e.g. means) or other basic estimates (e.g. regression coefficient) AND variation (e.g. standard deviation) or associated estimates of uncertainty (e.g. confidence intervals)
☐	☒	For null hypothesis testing, the test statistic (e.g. *F*, *t*, *r*) with confidence intervals, effect sizes, degrees of freedom and *P* value noted<br>*Give P values as exact values whenever suitable.*
☒	☐	For Bayesian analysis, information on the choice of priors and Markov chain Monte Carlo settings
☒	☐	For hierarchical and complex designs, identification of the appropriate level for tests and full reporting of outcomes
☒	☐	Estimates of effect sizes (e.g. Cohen's *d*, Pearson's *r*), indicating how they were calculated

Our web collection on statistics for biologists contains articles on many of the points above.

Software and code

Policy information about availability of computer code

Data collection	10x Genomics Cell Ranger (3.0.1 version)<br>BD FACSDiva Software (6.1.3 version)
Data analysis	The following commercial software was used in this study:<br>FlowJo v7.6.5, Metascape (online), Circos (online), inForm 2.4.10, kmplot (online), GSEA (online)<br>R (v3.5.1) with packages Seurat_3.0.2, monocle_2.10.1, WGCNA_1.69, scTHI_3.11, SCENIC_1.1.0, pheatmap_1.0.10, pyscenic_0.9.19, limma_3.36.3, RColorBrewer_1.1-2, Matrix_1.2-15, ggplot2_3.1.0

For manuscripts utilizing custom algorithms or software that are central to the research but not yet described in published literature, software must be made available to editors/reviewers. We strongly encourage code deposition in a community repository (e.g. GitHub). See the Nature Research guidelines for submitting code & software for further information.

Data

Policy information about availability of data

All manuscripts must include a data availability statement. This statement should provide the following information, where applicable:

- Accession codes, unique identifiers, or web links for publicly available datasets
- A list of figures that have associated raw data
- A description of any restrictions on data availability

The gene expression files for scRNA-seq are available at the Gene Expression Omnibus database (GES145370).

Field-specific reporting

Please select the one below that is the best fit for your research. If you are not sure, read the appropriate sections before making your selection.

[x] Life sciences [ ] Behavioural & social sciences [ ] Ecological, evolutionary & environmental sciences

For a reference copy of the document with all sections, see nature.com/documents/nr-reporting-summary-flat.pdf

Life sciences study design

All studies must disclose on these points even when the disclosure is negative.

Sample size	A total of twenty-eight pairs of samples (tumor and adjacent tissue) were analyzed based on the tissue availability. No samples size calculation was performed due to practical constraints of patient recruitment and sample collection.
Data exclusions	No data exclusion except quality controls when processing the raw scRNA-seq data. Low-quality cells (<400 genes/cell and >10% mitochondrial genes) were excluded.
Replication	As a result, 80,787 cells were included in downstream scRNA-seq analyses. For immunohistochemistry, 11 pairs of samples were stained and similar staining results was observed in over 3 visual fields. For FACS analysis, twenty-one pairs of samples were analyzed and more than 3 samples were tested for each panel. For in vitro blocking experiments, each design was repeated at least three times.<br>All replications were successful, and the detailed information was provided in corresponding figure legends.
Randomization	The patients with esophageal squamous cell carcinoma were recruited randomly in this study.
Blinding	Not applicable since there was no specific grouping.

Reporting for specific materials, systems and methods

We require information from authors about some types of materials, experimental systems and methods used in many studies. Here, indicate whether each material, system or method listed is relevant to your study. If you are not sure if a list item applies to your research, read the appropriate section before selecting a response.

Materials & experimental systems

n/a	Involved in the study	
[ ]	[x]	Antibodies
[x]	[ ]	Eukaryotic cell lines
[x]	[ ]	Palaeontology
[x]	[ ]	Animals and other organisms
[ ]	[x]	Human research participants
[x]	[ ]	Clinical data

Methods

n/a	Involved in the study	
[x]	[ ]	ChIP-seq
[ ]	[x]	Flow cytometry
[x]	[ ]	MRI-based neuroimaging

Antibodies

Antibodies used

Flow antibodies:
CD117 (c-kit)-PE/Cyanine7 (Clone: 104D2, Catlog#: 313212; Biolegend)
CD11b-APC (Clone: CBRM1/5, Catlog#: 17-0113-42; eBioscience)
CD11c-PerCP/Cyanine5.5 (Clone: 3.9, Catlog#: 301623; Biolegend)
CD11c-FITC (Clone: 3.9, Catlog#: 11-0116-42; eBioscience)
CD138 (Syndecan-1)-PE/Cyanine7 (Clone: DL-101, Catlog#: 25-1389-42; eBioscience)
CD14-PE/Cyanine7 (Clone: M5E2, Catlog#: 561385; BD Pharmingen)
CD15 (SSEA-1)-APC/Cyanine7 (Clone: W6D3, Catlog#: 323048; Biolegend)
CD159a (NKG2A)-FITC (Clone: REA110, Catlog#: 130-113-565; Miltenyi Biotec)
CD163-PerCP/eFluor 710 (Clone: eBioGHI/61 (GHI/61), Catlog#: 46-1639-42; eBioscience)
CD19-PerCP/Cyanine5.5 (Clone: HIB19, Catlog#: 302230; Biolegend)
CD197 (CCR7)-PE/Cyanine7 (Clone: G043H7, Catlog#: 353225; Biolegend)
CD206 (MMR)-Alexa Fluor 488 (Clone: 19.2, Catlog#: 53-2069-42; eBioscience)
CD235ab-FITC (Clone: HIR2, Catlog#: 306610; Biolegend)
CD25-FITC (Clone: M-A251, Catlog#: 356106; Biolegend)
CD274 (PD-L1)-BV421 (Clone: MIH1, Catlog#: 563738; BD Horizon)
CD279 (PD-1)-PerCP/Cyanine5.5 (Clone: EH12.2H7, Catlog#: 329913; Biolegend)
CD3-APC (Clone: HIT3a, Catlog#: 300312; Biolegend)

CD3-PerCP/Cyanine5.5 (Clone: HIT3a, Catlog#: 300327; Biolegend)
CD4-PerCP/Cyanine5.5 (Clone: OKT4, Catlog#: 317428; Biolegend)
CD4-PerCP/Cyanine5.5 (Clone: A161A1, Catlog#: 357409; Biolegend)
CD45-eFluor 450 (Clone: 2D1, Catlog#: 48-9459-42; eBioscience)
CD45-APC (Clone: HI30, Catlog#: 560973; BD Pharmingen)
CD45-APC/Cyanine7 (Clone: HI30, Catlog#: 304014; Biolegend)
CD45-PerCP/Cyanine5.5 (Clone: 2D1, Catlog#: 368503; Biolegend)
CD56 (NCAM)-APC/Cyanine7 (Clone: HCD56, Catlog#: 318332; Biolegend)
CD56 (NCAM)-PE/Cyanine5.5 (Clone: CMSSB, Catlog#: 35-0567-42; eBioscience)
CD63 (LAMP-3)-Alexa Fluor 647 (Clone: H5C6, Catlog#: 561983; BD Pharmingen)
CD66b-FITC (Clone: G10F5, Catlog#: 305104; Biolegend)
CD68-FITC (Clone: Y1/82A, Catlog#: 333805; Biolegend)
CD68-APC/Cyanine7 (Clone: Y1/82A, Catlog#: 333822; Biolegend)
CD68-PE (Clone: Y1/82A, Catlog#: 12-0689-42; eBioscience)
CD8-APC/Cyanine7 (Clone: SK1, Catlog#: 344714; Biolegend)
CD83-APC/Cyanine7 (Clone: HB15e, Catlog#: 305330; Biolegend)
FOXP3-eFluor 450 (Clone: 236A/E7, Catlog#: 48-4777-42; eBioscience)
FOXP3-PE (Clone: 259D/C7, Catlog#: 560046; BD Pharmingen)
HLA-ABC-PE (Clone: W6/32, Catlog#: 12-9983-42; eBioscience)
HLA-DR-eFluor 450 (Clone: L243, Catlog#: 48-9952-42; eBioscience)
HLA-DR-PE (Clone: L243, Catlog#: 307605; Biolegend)
IDO-FITC (Clone: eyedio, Catlog#: 11-9477-42; eBioscience)
IFN-γ-BV421 (Clone: B27, Catlog#: 562988; BD Horizon)
IL-1 RII-APC (Clone: 34141, Catlog#: FAB663A; R&D Systems)
TNF-a-APC (Clone: MAb11, Catlog#: 17-7349-82; eBioscience)
LILRB1 (Clone: Polyclonal, Catlog#: AF2017; R&D Systems)
Donkey Anti-Goat IgG-NorthernLights 637 (Catlog#: NL002; R&D Systems)
Stimulation or blocking antibodies:
CD3 monoclonal antibody (Clone: OKT3; Catlog#:16-0037-81, eBioscience)
CD28 monoclonal antibody (Clone:CD28.2; Catlog#: 16-0289-81, eBioscience)
IL-1 RII monoclonal antibody (Clone:34141; Catlog#: MAB663-100, R&D Systems)
LILRB1 (Clone: Polyclonal, Catlog#: AF2017; R&D Systems)
Mouse IgG2a isotype control (Clone: Ebm2a, Catlog#: 16-4724-82, Invitroen)
Multi-color immunohistochemistry antibodies:
CD4 (Clone: EPR6855, Host:R, Catlog#: ab181724; Abcam)
FOXP3 (Clone: Polyclonal, Host:M, Catlog#: ab22510; abcam)
LILRB1 (Clone: EPR11256, Host:R, Catlog#: ab249583; Abcam)
CD68 (Clone: KP1, Host:M, Catlog#: ab955; Abcam)
CD11c (Clone: EP1347Y, Host:R, Catlog#: ab216655; Abcam)
IDO (Clone: SP260, Host:R, Catlog#: ab245737; Abcam)
PD-L1 (Clone: E1L3N, Host:R, Catlog#: 13684S; CST)
LAMP3 (Clone: Polyclonal, Host:R, Catlog#: ab111090; Abcam)
Immunohistochemistry antibodies:
CD20 (Clone: L26, Host:M, Catlog#: NCL-L-CD20-L26; LEICA)
CD3 (Clone: LN10, Host:M, Catlog#: NCL-L-CD3-565; LEICA)
Myeloperoxidase (MPO) (Clone: EP151/SP72, Host:R, Catlog#: 0405; Long island)
CD56 (Clone: 56C04, Host:M, Catlog#: 0148; Long island)
CD68 (Clone: KP1/PG-M1, Host:M, Catlog#: 0160; Long island)

Validation

Available on the manufacturers' websites: http://www.thermofisher.com; http://biolegend.com; http://abcam.com; https://www.miltenyibiotec.com; https://www.bdbiosciences.com; https://www.rndsystems.com; https://www.cellsignal.cn; www.longislandab.com; https://www.leicabiosystems.com

Human research participants

Policy information about studies involving human research participants

Population characteristics	The detailed clinical information of these patients was summarized in Supplementary Table 1. The number of patients are 28 (7 scRNA-seq, 21 Flow analysis); age ranged from 53 to 81 (median 67); only 2 females; pathology stage range from IIA to IVA.<br>PBMC from 30 healthy controls was used for macrophage differentiation and antibody blocking experiments. Healthy volunteers age ranged from 34 to 62 (median 46); 15 females;
Recruitment	The patients who were pathologically diagnosed with ESCC were enrolled in this study. None of the patients had been treated with chemotherapy, radiation, or any other anti-tumor medicines prior to tumor resection. Samples were randomly collected from the Xinhua and Ruijin Hospital, Shanghai Jiaotong University School of Medicine between 2019 and 2020. The adjacent normal tissues were at least 5 cm from the tumor tissues.<br>Only patients who meet the surgical indications will be collected and analyzed, which may cause bias.<br>Healthy volunteers who showed normal blood routine and biochemical indicators after physical examination and no history of other major diseases were recruited.
Ethics oversight	Prior to participation, written informed consent was obtained from all subjects. All studies were performed in accordance with the Declaration of Helsinki. The study was approved by the Research Ethics Board of the Xinhua and Ruijin Hospitals, Shanghai Jiao Tong University School of Medicine.

Note that full information on the approval of the study protocol must also be provided in the manuscript.

Flow Cytometry

Plots

Confirm that:

- [x] The axis labels state the marker and fluorochrome used (e.g. CD4-FITC).
- [x] The axis scales are clearly visible. Include numbers along axes only for bottom left plot of group (a 'group' is an analysis of identical markers).
- [x] All plots are contour plots with outliers or pseudocolor plots.
- [x] A numerical value for number of cells or percentage (with statistics) is provided.

Methodology

Sample preparation	1. CD45+ cells from the tumor and normal tissues were isolated by mincing the freshly obtained surgical specimens into 1-mm cubic pieces, followed by enzymatic digestion using 0.1% collagenase IV, 0.002% DNAse I, and 0.01% hyaluronidase, and were incubated on a rocker for 20-40 min at 37°C. The digested tissues were then passed through a 40 μm cell strainer and washed twice with PBS prior to staining.<br>2. Immune cells were stained at 1×106 cells per ml with surface antibodies for 30 min at 4°C, and then washed and resuspended in 200 μL FACS buffer. PI was added 5 min before flow cytometry sorting or detection.<br>3. For intracellular cytokine staining, cells were stimulated with cell stimulation cocktail plus protein transport inhibitors (eBioscience) for 5 h. Then, the cells were fixed and permeabilized with Cytofix/Cytoperm buffer, and intracellular cytokines were stained with antibodies and isotype control according to the manufacturer's instructions.<br>4. For nuclear molecule staining, cells were fixed and permeabilized with Foxp3 / Transcription Factor Staining Buffer Set (eBioscience) follow the instructions.
Instrument	FACS Aria II Cell Sorter (BD Biosciences) (only for sorting); FACS Canto II instrument (BD Bioscience)
Software	BD FACSDiva Software v6.1.3, FlowJo v7.6.5
Cell population abundance	CD45+CD235- PI- cells, CD4+CD25- Teff cells, CD4+CD25hiCD127- Treg cells were sorted and the purity was routinely > 95%. After sorting, cells were resuspended at a 1×106 cells/ml concentration in FACS buffer and the viability was higher than 90%.
Gating strategy	The FSC/SSC gating strategy was used to exclude cell debris and doublets. In the sorting process, CD45-APC, CD235-FITC, PI were used to exclude apoptotic cells (PI+) and erythrocytes (CD235+) and ensure immune cells (CD45+) purity. In flow analysis, different combinations of marker panels were design for various immune subsets, the gating strategies are shown in Supplementary Fig.12.

- [x] Tick this box to confirm that a figure exemplifying the gating strategy is provided in the Supplementary Information.
